# Effect of implementing an acute myocardial infarction guideline on quality indicators

**DOI:** 10.1590/S1679-45082013000300016

**Published:** 2013

**Authors:** Marcia Makdisse, Marcelo Katz, Alessandra da Graça Corrêa, Luciano Monte Alegre Forlenza, Marco Antonio Perin, Fábio Sândoli de Brito, Teresa Cristina Dias Cunha Nascimento, Ivanise Maria Gomes, Marcelo Franken, Marcos Knobel, Antonio Eduardo Pereira Pesaro, Oscar Fernando Pavão dos Santos, Miguel Cendoroglo, Claudio Luiz Lottenberg

**Affiliations:** 1 Hospital Israelita Albert Einstein, São Paulo, SP, Brazil.

**Keywords:** Practice guidelines as topic, Quality indicators, health care, Myocardial infarction, Quality of health care

## Abstract

**Objective:**

To evaluate the compliance rates to quality of care indicators along the implementation of an acute myocardial infarction clinical practice guideline.

**Methods:**

A clinical guideline for acute myocardial infarction was introduced on March 1^st^, 2005. Patients admitted for acute myocardial infarction from March 1^st^, 2005 to December 31^st^, 2012 (n=1,431) were compared to patients admitted for acute myocardial infarction before the implementation of the protocol (n=306). Compliance rates to quality of care indicators (ASA prescription on hospital admission and discharge, betablockers on discharge and door-to-balloon time) as well as the length of hospital stay and in-hospital mortality were compared before and after the implementation of the clinical guideline.

**Results:**

The rates of ASA prescription on admission, on discharge and of betablockers were higher after guideline implementation: 99.6% *versus* 95.8% (p<0.001); 99.1% *versus* 95.8% (p<0.001); and 95.9% *versus* 81.7% (p<0.001), respectively. ASA prescription rate increased over time, reaching 100% from 2009 to 2012. Door-to-balloon time after *versus* before implementation was of 86(32) minutes *versus* 93(51) (p=0.20). The length of hospital stay after the implementation *versus* before was of 6(6) days *versus* 6(4) days (p=0.34). In-hospital mortality was 7.6% (before the implementation), 8.7% between 2005 and 2008, and 5.3% between 2009 and 2012, (p=0.04).

**Conclusion:**

The implementation of an acute myocardial infarction clinical practice guideline was associated with an increase in compliance to quality of care indicators.

## RESUMO

**Objetivo:**

Avaliar a adesão aos indicadores de qualidade assistencial ao longo da implementação de um protocolo assistencial de infarto agudo do miocárdio.

**Métodos:**

Em 1º de março de 2005 foi implementado o protocolo assistencial de infarto agudo do miocárdio. Foram selecionados pacientes admitidos de 1º de março de 2005 a 31 de dezembro de 2012 (n=1.431). Para comparação, utilizamos os dados de pacientes admitidos por infarto na fase pré-protocolo (n=306). Comparamos a taxa de adesão aos indicadores (taxa de prescrição de AAS na admissão hospitalar e na alta hospitalar, betabloqueador na alta e tempo porta-balão) entre as fases pré e pós-implementação do protocolo, além de tempo de permanência hospitalar e mortalidade intra-hospitalar nas diferentes fases.

**Resultados:**

As taxas de prescrição de AAS na admissão e na alta hospitalar, e de betabloqueador foram maiores na fase pós *versus* a pré-implementação do protocolo: 99,6% *versus* 95,8% (p<0,001); 99,1% *versus* 95,8% (p<0,001) e 95,9% *versus* 81,7% (p<0,001), respectivamente. A taxa de prescrição de AAS aumentou ao longo da implementação do protocolo, atingindo 100% de 2009 a 2012. O tempo porta-balão pós *versus* pré foi de 86(32) minutos *versus* 93(51), respectivamente (p=0,20). O tempo de permanência hospitalar foi semelhante na fase pré *versus* pós-protocolo: 6(6) dias *versus* 6(4) dias (p=0,34). A mortalidade intra-hospitalar foi de 7,6% no pré-protocolo, 8,7% entre 2005 e 2008 e 5,3% entre 2009 e 2012 (p=0,04).

**Conclusão:**

A implementação do protocolo assistencial refletiu-se na maior adesão aos indicadores de qualidade.

## INTRODUCTION

In recent years the diagnosis and treatment of cardiovascular diseases have been improved as a consequence of contemporary knowledge and the incorporation of new technologies. Despite that, the implementation of clinical practice guidelines in health care services is still far from what would be expected. As a result, the health care system performance is lower than it should be, compromising patients' safety and needs^(1)^. In 1999 the Institute of Medicine published staggering data on errors occurring in the care process. This report stated that between 44 and 98 thousand deaths occurred in the United States due to errors in processes related to patient care. The number of damages resulting from errors was even greater reaching 1 million injuries each year^(2,3)^. As a result, medical societies launched initiatives aimed at improving the quality of health care, thus raising safety and reducing unfavorable outcomes during hospitalization. The Agency for Healthcare Research and Quality (AHRQ) has defined care quality as doing the right thing, at the right time, in the right way, for the right person - and having the best results possible^(1,4)^.

The association between evidence-based medicine (EBM), which focused more on stimulating clinical decisions based on best evidences available (“doing the right things”), and the Clinical Quality Improvement movement which focused more on the use of EBM knowledge to change processes related to recurrent problems within the systems of care (“doing things right”), enables an integrated and complementary view that can improve care quality (“to do the right things right”)^(5)^.

A number of indicators have been recommended to measure the quality of care delivered to patients with acute myocardial infarction (AMI). However, indicators monitoring per se does not assure the incorporation of evidence-based therapy on clinical practice. Therefore, it is necessary to know and refine the process of care, engage health care professionals and select the most suitable quality improvement tools for each context^(6)^_._

Several clinical quality improvement projects have used, as part of their strategy to improve care and implement EBM, the four steps approach (PDCA-Plan-Do-Check-Act or PDSA-Plan-Do-Study-Act) which involve: definition of priorities (Plan), implementation of clinical guidelines (Do), measurement of performance (Check/Study) and improvement of performance (Act)^(7–10)^.

When dealing with complex systems, such as health care services, which involve different stakeholders, multifaceted strategies combining at least two methods, such as education, facilitation, audit, benchmarking, feedback, benefits, among others, enhance the likelihood of success. Besides, these strategies should be focused on both clinical and administrative staff^(11)^.

At *Hospital Israelita Albert Einstein* (HIAE) the AMI clinical guideline was implemented on March 2005. Since then AMI quality indicators have been monitored during hospitalization. Medical literature lacks meaningful robust evidence on clinical quality improvement, and few publications have evaluated the effect of multifaceted strategy on compliance to quality indicators and on clinical outcomes^(12)^.

## OBJECTIVE

To assess the rate of compliance to quality indicators after the implementation of an acute myocardial infarction clinical practice guideline.

## METHODS

### Population and management of the acute myocardial infarction clinical practice guideline

The AMI guideline was implemented on March 1st, 2005. The main interventions are described on chart 1.

**Chart 1:** Interventions used on the acute myocardial infarction clinical practice guideline implementation

Intervention	Actions	Target
Clinical guideline design	Meetings with both employed and self-employed physicians;	Medical and multidisciplinary staff
	Customizing the guideline to suit different Emergency Departments (ED) within HIAE, including criteria to define reperfusion therapy strategy (either primary angioplasty or fibrinolysis) and flowcharts to other recommended therapies.	
Organizing: to evaluate and promote changes in the process of care	To identify an ED cardiologist to facilitate guideline implementation process, with adouble report to the emergency and Cardiology Departments (Hybrid physician);	Medical and multidisciplinary staff, and managers
	Development of a new cardiac triage tool:	
	Identification of patients with priority for ECG;	
	AMI code : simultaneous activation of transport, catheterization lab team, anesthesiologist and nurse case manager;	
	Initial treatment conducted by the ED on-duty cardiologist;	
	On-duty ED cardiologists to support the satellite units on the treatment decision (conservative, fibrinolysis or primary angioplasty)	
Guideline Dissemination	The Guideline publication in Medical Suite – a virtual platform to communicate with clinical staff	Medical and multidisciplinary staff, and managers
	Educational meetings with cardiologists and multidisciplinary team;	
	Partnership with opinion leaders.	
Patient education	Brochures with information about the AMI, its risk factors and medications.	Patients
Auditing indicators	Recruiting a nurse case manager;	Medical and multidisciplinary staff
	Selecting indicators;	
	Creating a database;	
	Conducting daily rounds to audit the indicators.	
Feedback	To the multidisciplinary staff directly involved with AMI patients care : daily report highlighting the status of compliance to indicators (by e-mail);	Medical and multidisciplinary staff, and managers
	To the on duty and self-employed physician in charge of the patients: feedback on non-conformities and request to document contraindications and/or conditions for non-prescription in the medical record;	
	To the self-employed cardiology staff (partnership with the Medical Practice Division): Letter informing individual performance elated to compliance to AMI quality indicators, comparing with the mean performance achieved by their peers (to 100% of cardiologists); Personal feedback to 20 to 30% of cardiologists (in charge of 80% of cardiac admissions);	
	To managers: monthly report to managers of satellite units, coronary care unit and ICU on the performance concerning the quality indicators; report to the HIAE medical director; bimonthly report to the SBIBAE Advisory and Executive Board.	
Incentive Program	Compliance to AMI clinical guideline indicators were included as credits for the institutional incentive program directed to the self-employed staff	Medical staff
Meetings to adapt the guideline	Meetings to discuss cases of non-conformity, to adjust processes and design new actions. The meetings were headed by the guideline management team (hybrid physicians and nurse case manager) and were attended by the ED, interventional cardiology and patient transportation staff.	Medical and multidisciplinary, and managers
Disclosure of results	Presentation compliance to quality indicators and outcomes were shared at scientific meetings and forum for specialists.	Medical and multidisciplinary staff, managers and patients
	Publication of indicators at the institutional homepage http://www.einstein.br/qualidade-seguranca-do-paciente/Paginas/indicadores-assistenciais.aspx (available from 2008)	
	Publication of indicators in the annual report for specialists (available both in printed and electronic format)	
Report to external agencies	Reports to ANAHP and The Joint Commission (during reaccreditation processes)	

HIAE: *Hospital Israelita Albert Einstein*; ECG: electrocardiogram; ICU: intensive care unit; ANAHP: National Association of Private Hospitals.

Inpatients with AMI were identified during daily rounds at admission units and through medical records review in cases of activation of the AMI code, also from the daily report sent by the clinical laboratory including values for cardiac troponin and from reports of the institutional epidemiology and statistics service.

Since the guideline was implemented, a prospective database was set up to assure the record of quality of care indicators and clinical outcomes. Information on admitted patients with AMI was included in the database (according to the ICD-10 discharge diagnosis for AMI, and institutional epidemiology service) for subsequent comparison.

The criteria recommended by the Joint Commission were used to identify eligible and non-eligible patients in order to generate quality indicators. These criteria include indication to therapies, the presence of contraindications or conditions in medical record for the non-prescription of the therapy, such as patients' refusal, cardiorespiratory arrest, among others.

We excluded patients younger than 18 years old, those with a hospital stay longer than 120 days, clinical trial participants and also patients transferred from other services. In addition, patients who in the first 24 hours needed palliative care only, were transferred or requested hospital discharged, and/or those who died were also excluded^(13)^. All patients admitted using the guideline had their eligibility or non-eligibility confirmed by the nurse case manager.

The AMI guideline database was approved by the Ethical and Research Committee of *Hospital Israelita Albert Einstein* (HIAE), São Paulo (Einstein Acute Myocardial Infarction Registry, Research project nº 1,282-10).

Patients were selected at admission in the Emergency Room (ER) at the following units: *Morumbi*, *Alphaville*, *Ibirapuera* and *Perdizes* from March 1^st^ 2005 to 31^st^ December 2012. The pre-guideline evaluation used data from database of AMI patients admitted in this first phase. Periods were classified in years. Pre-guideline phase occurred from previous years to 2005 and post-guideline phase from 2005 to 2012. This latter phase was divided into guideline maturity (2005–2008) and established guideline (2009-2012).

### Quality indicators

For comparison with other institutions the quality indicators were selected based on national and international guidelines for AMI, recommendations from organization specialized in providing guidance and auditing quality of care^(13–16)^. Quality indicators included were: rate of ASA prescription at hospital admission and discharge, β-blockers on discharge and door-to-balloon time.

To measure the rate of drug prescription the following formula was used:


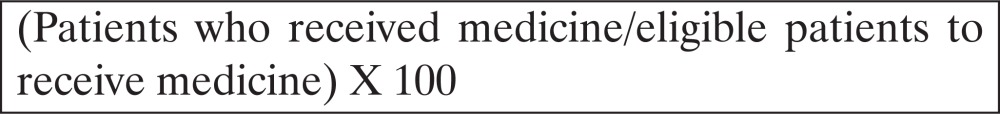


The median door-to-balloon time was calculated in minutes only for AMI patients with ST-segment elevation, and who were eligible to reperfusion therapy, as the time between admission in the ER and the performance of the primary angioplasty with the opening of the artery responsible for the AMI at the catheterization laboratory.

### Clinical outcomes

Clinical outcomes included in the analysis were length of hospital stay and in-hospital mortality. All deaths were considered in the analysis.

### Statistical analysis

Data are presented in means±standard deviation or median and interquartile variation for continuous variables, and as absolute and relative frequencies for categorical variables. Sample comparison was made using Student's t test or Mann-Whitney test for continuous variables, and the chi-squared test for categorical variables. p<0.05 was considered statistically significant.

## RESULTS

The guideline included data on 1,431 patients with AMI admitted at the ER of the four hospital units, being 89.77% at *Morumbi*, 5.7% at *Alphaville*, 2.9% at *Ibirapuera* and 1.7% at *Perdizes*. Data on 306 patients admitted at *Morumb*i ER Unit from 2002 to 2005 (pre-protocol) was used for comparison. Table 1 describes the clinical characteristics of patients in the different phases of the project.

**Table 1:** Pre *versus* post-guideline clinical characteristics of patients

Clinical characteristic	Pre-guideline (n=306)	Post-guideline (n=1,431)	p value
Men (%)	68	70	0.48
Age (years)	66±14	68±15	0.11
DM (%)	27	33	0.07
Hypertension (%)	51	59	0.007
Smoking (%)	27	19	0.003
Dyslipidemia (%)	21	37	<0.001
ST segment elevation AMI (%)	63	38	<0.001

DM: diabetes mellitus; AMI: acute myocardial infarction.

### Quality indicators

**Figure 1:**
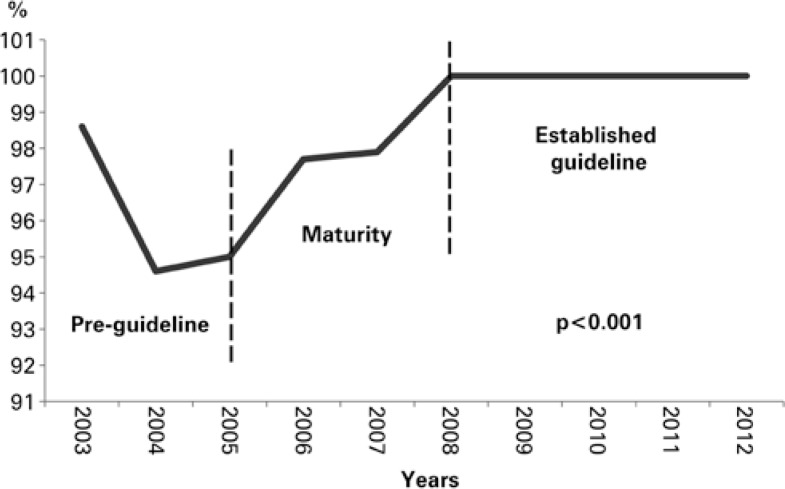
Rate of prescription of acetylsalicylic acid on discharge based on guideline implementation phase

The rate of ASA prescription at admission and discharge were higher at post-guideline implementation compared with pre-implementation; 99.6% *versus* 95.8% (p<0.001) and 99.1% *versus* 95.8% (p<0.001), respectively. The rate of ASA prescription on discharge showed a growing tendency, reaching 100% after three years of the implementation phase (Figure 1).

The rate of β-blocker prescriptions on discharge was higher in post-guideline compared with pre-guideline phase: 95.9% *versus* 81.7% (p<0.001).

The median variation of door-to-balloon time measured in patients referred to recanalization therapy was not significant after the guideline implementation (pre *versus* post, median and interquartile variation): 93(51) minutes *versus* 86(32) minutes (p=0.20).

### Clinical outcomes

The median length of hospital stay was similar for both pre and post-implementation phase: 6(6) days *versus* 6(4) days (p=0.34).

**Figure 2:**
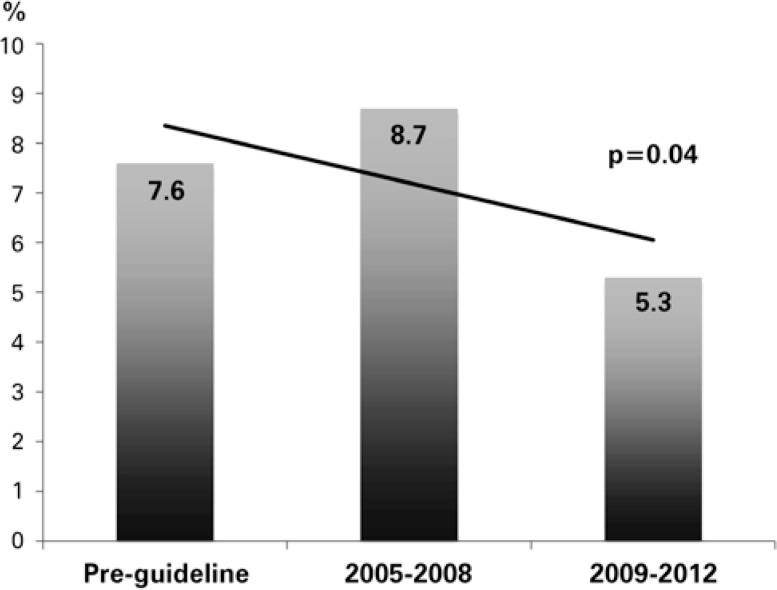
Rate of in-hospital mortality during guideline implementation phases

The analysis of mortality during the implementation periods showed a decreased of in-hospital deaths in the last four years (Figure 2).

## DISCUSSION

In this study, compliance to AMI quality of care indicators increased over time and, although the length of hospital stay did not change, in-hospital mortality dropped in the last years.

We observed a higher compliance rate to the guideline after three years of its implementation suggesting the need of a maturing phase in which actions are progressively accepted and incorporated into clinical practice. These findings are consistent with literature that points out to a behavioral change - described as a process that involves several phases (precontemplation, contemplation, preparation and action) - that could be accelerated by the use of adequate interventions^(17)^.

Clinical guidelines implemented at institutions had been associated with higher compliance to quality indicators resulting in lower clinical practice variability. However, the majority of studies evaluated the effect of interventions only a few months after its implementation, and long-term effects have not been much explored in the literature^(18)^. Ultimately, the final goal of evidence-based guideline implementation should be the improvement of clinical outcomes and healthcare costs.

The decision for a multifaceted strategy, customized for the hospital context may have contributed to the improved compliance to quality indicators observed in our analysis. According to the literature, the most frequently used multifaceted interventions are educational materials (48%), educational meetings (41%), remembering notes (31%), audits and feedback (24%)^(11)^. These interventions were used in the setting of this study.

In the context of self-employed clinical staff, among whom the protocol was implemented, we believe that audits and feedbacks with the inclusion of individual performances as part of the Institutional Incentive Program was fundamental to improve results. Such actions reinforce the institutional commitment to guarantee quality and safety of care delivery to the patient. Additionally, rewarding physicians for their performance on quality metrics and outcomes rather than solely on their volume of procedures overcomes one of the perceived barriers to guideline implementation which is volume-based incentive^(19)^.

However, a systematic review with 118 studies published at Cochrane Library^(20)^ showed an inconsistent association between feedback and compliance improvement, regardless of interventions used (ranging from a reduction of 16% up to an increase of 70% in compliance rate). The lower the baseline compliance rate and the higher the intensity of the feedback, greater results were observed. The review did not consider the context in which these interventions were implemented^(20)^.

Importantly, the level of organization of the self-employed staff was a key issue to increase the compliance. Since 2003 cardiologists have regular scientific meetings organized by staff opinion leaders. This communication route along with Cardiology Forums, created a few years later, was fundamental to involve the physician's opinion leaders in the guideline design and implementation. The identification and commitment of such leaders represent a strategy that has been used to facilitate clinical guidelines implementation^(21)^.

The fact that the guideline was targeted at cardiologists may have contributed to an easier implementation. A study published in the New England Healthcare Institute^(19)^ pointed out that cardiologists showed higher adherence to clinical guidelines than others specialists. In a scale of change that goes from “pre-contemplation” to “action/ adherence”, 70% of cardiologists were in the last phase compared with 47% of general practitioners, 34% of other specialists and 25% of orthopedists. Cardiologists also reported to find fewer barriers to guidelines implementation, such as disagreement with recommendations, diagnostic uncertainty, and lack of technology, among others.

From the organizational point of view, there are evidences that factors, such as leadership support, interprofessional collaboration, sharing of beliefs and values also influence adherence to clinical guidelines^(22)^. The continuous search for quality improvement is one of the values of our institution, and because our hospital was the first in Latin America to be accredited by The Joint Commission it certainly contributed to create a quality and patient safety culture. In 2007 the third year of the guideline implementation, quality and patient safety became part of the institution strategy, requiring a strict leadership commitment in order to avoid risks in care delivery. This strategy included benchmarking with high-performing institutions, known as positive deviants, in order to create opportunities to identify and disseminate new actions to improve quality of care^(23)^.

The implementation of the clinical guideline brought benefits beyond those related to improving the compliance to quality indicators; it provided higher integration among care teams that become more aligned and coordinated, particularly because the guideline implementation is not an isolated or specific action, but a continuum involving design and redesign of care processes in order to correct and improve them bearing in mind the lessons learned from the earlier phase based on quality cycles (Plan-Do- Study-Act)^(24)^.

### Limitations

This study had some limitations. Patients included in the pre-implementation phase did not represent the total of admitted patients for AMI from 2002 to 2005. Thus, it is likely that this may have caused a higher proportion of AMI patients with ST segment elevation in the pre-implementation phase. We believe that this fact is not the main driver for the difference observed in the compliance rates. It also did not compromise the analysis of door-to-balloon time as only eligible patients were considered. In addition, we could not identify the efficacy of one specific intervention in improving the adherence to indicators. Perhaps, all interventions acting together provided the improvement observed in our analysis.

## CONCLUSION

The implementation of an acute myocardial infarction clinical practice guideline, based on multifaceted intervention strategy, was associated with an increase in compliance to quality of care indicators.
